# Association of Energy Intake With the Lack of in-Person Review of Household Dietary Records: Analysis of Japan National Health and Nutrition Surveys From 1997 to 2011

**DOI:** 10.2188/jea.JE20150048

**Published:** 2016-02-05

**Authors:** Nayu Ikeda, Nagako Okuda, Megumi Tsubota-Utsugi, Nobuo Nishi

**Affiliations:** 1 Center for International Collaboration and Partnership, National Institute of Health and Nutrition, National Institutes of Biomedical Innovation, Health and Nutrition, Tokyo, Japan; 1 国立健康・栄養研究所 国際産学連携センター; 2 Department of Health and Nutrition, University of Human Arts and Sciences, Saitama, Japan; 2 人間総合科学大学 健康栄養学科

**Keywords:** total energy intake, National Health and Nutrition Survey, semi-weighed dietary records, in-person review, 総エネルギー摂取量, 国民健康・栄養調査, 半定量法による食事記録, 対面確認

## Abstract

**Background:**

National surveys have demonstrated a long-term decrease in mean energy intake in Japan, despite the absence of a decrease in the prevalence of overweight and obesity. We aimed to examine whether total energy intake of survey respondents is associated with completion of an in-person review of dietary records and whether it affects the trend in mean energy intake.

**Methods:**

We pooled data from individuals aged 20–89 years from the National Nutrition Surveys of 1997–2002 and the National Health and Nutrition Surveys of 2003–2011. We conducted a linear mixed-effects regression to estimate the association between total energy intake and the lack of an in-person review of semi-weighed household dietary records with interviewers. As some respondents did not have their dietary data confirmed, we used regression coefficients to correct their total energy intake.

**Results:**

Compared with respondents completing an in-person review, total energy intake was significantly inversely associated with respondents not completing a review across all sex and age groups (*P* < 0.001). After correction of total energy intake for those not completing a review, mean energy intake in each survey year significantly increased by 2.1%–3.9% in men and 1.3%–2.6% in women (*P* < 0.001), but the decreasing trend in mean energy intake was sustained.

**Conclusions:**

Total energy intake may be underestimated without an in-person review of dietary records. Further efforts to facilitate completion of a review may improve accuracy of these data. However, the increasing proportion of respondents missing an in-person review had little impact on the decreasing mean caloric intake.

## 要約

**背景：:**

国民健康・栄養調査の結果によると、平均エネルギー摂取量は長期に渡り低下傾向にあるが、過体重と肥満の有病率の減少傾向は示されていない。本研究は、国民健康・栄養調査において調査員と被調査者が対面で行う食事記録の確認の有無が、総エネルギー摂取量と関連するか、また平均エネルギー摂取量の減少傾向に影響を与えているかについて検討した。

**方法：:**

国民栄養調査（平成9年～平成14年）および国民健康・栄養調査（平成15年～平成23年）の20歳から89歳の個人のデータを統合した。総エネルギー摂取量と、半定量法による世帯単位の食事記録に関する被調査者との対面確認が欠落していることとの関連について、線形混合効果モデルによる推定を行った。食事記録が確認されていない被調査者については、総エネルギー摂取量を回帰係数で調整した。

結果：対面確認を完了した被調査者と比較して、確認を完了していない被調査者の総エネルギー摂取量は、すべての性・年齢階級において有意な負の相関を示した（P値<0.001）。対面確認を完了していない被調査者の総エネルギー摂取量の補正により、各調査年における平均エネルギー摂取量は男性では2.1%から3.9%、女性では1.3%から2.6%、有意に上昇したが（P値<0.001）、平均エネルギー摂取量の減少傾向には変化がなかった。

**結論：:**

食事記録の対面確認を完了していないことにより、総エネルギー摂取量は過少に推定される可能性がある。今後、対面確認の完了を促進する取り組みによって、これらデータの精度の向上が見込まれる。一方で、対面確認を完了していない被調査者の割合の増加は、平均エネルギー摂取量の減少にほとんど影響を与えていない可能性が示唆された。

## INTRODUCTION

The prevalence of overweight and obesity has been growing worldwide for the past 30 years,^1^ making trends in mean energy intake of significant public health interest. A rise in caloric intake is a potential contributor to excess body weight, which can lead to morbidity and mortality from obesity-related non-communicable diseases.^2^ Therefore, monitoring of dietary energy consumption at the population level is essential for planning and evaluating programs for the prevention and control of disease burden on society.

In Japan, the prevalence of overweight and obesity in the population aged 20 years and older has increased among men from 18% in 1980 to 30% in 2010, while it has been stable at approximately 20% among women.^3^ The diet and nutritional status of the population of Japan has been monitored since 1947 through annual cross-sectional household surveys using nationally representative samples. These surveys have shown that the national average caloric intake increased after the end of the World War II, but started to gradually decrease in the early 1970s. In the 21st century, mean energy intake has continued to decrease among adults under 70 years, while it has remained constant for the older population.^3^ Although the absence of an increase in mean daily caloric intake has recently been reported from national dietary surveys in other industrialized countries,^4^^–^^7^ only Japan has found a long-term downward trend at the national level.

No previous study has investigated what accounts for the decrease in mean energy intake in parallel with the rising prevalence of overweight and obesity in Japan. One of the potential factors is a methodological issue: total energy intake may be underestimated for an increasing proportion of survey respondents who do not have their dietary records checked directly with interviewers. The Japanese survey adopts a semi-weighed household dietary record, in which representatives of participating households measure food and beverages consumed in the household over a single day and approximate distribution ratios of shared dishes across household members to obtain individual dietary intake.^8^ To ensure accuracy of the data, survey interviewers are required to conduct an in-person review of questionnaires with participants, either when interviewers visit participating households for collection of questionnaires or when survey respondents visit a site for a physical examination conducted as part of the survey on a later date. However, it is suspected that an increasing number of respondents avoid these opportunities for an in-person review, partly reflecting changes in social conditions, such as the emerging diversity of family life and changes in employment conditions.

Underreporting by survey respondents is a common and well-accepted source of systematic measurement error in dietary assessment.^9^ Previous studies have assessed underreporting of energy intake of individual respondents in national nutrition surveys that have used 24-hour recall and weighed dietary records.^10^^–^^14^ However, no study has examined whether the lack of an in-person review of semi-weighed household dietary records is associated with total energy intake estimated from the survey in Japan and whether it affects secular trends in mean energy intake of the population. Therefore, our objective was to examine the possibility of underestimation of total energy intake among survey respondents who do not participate in an in-person review of a household dietary questionnaire with a survey interviewer. We also explored whether trends in mean energy intake could change if all respondents had completed a review.

## MATERIALS AND METHODS

### Data

After excluding 5565 cases (4.2%) with missing data on variables used in this study, we pooled the individual-level data of 126 201 participants aged 20–89 years (58 290 men and 67 911 women) from National Nutrition Surveys from 1997 to 2002 and National Health and Nutrition Surveys from 2003 to 2011. We used the surveys from 1997–2011 because blood tests had been consistently conducted for participants aged 20–89 since 1997 and individual-level data were available up to 2011, when this study was implemented. In accordance with the Ethical Guidelines of Epidemiological Research,^15^ our study was exempted from the application of these guidelines because we used only information that had already been anonymized at the time of the study design.

Since 1947, the National Nutrition Survey has been implemented by the Japanese government to assess the nation’s health and nutritional status. The survey was redesigned in 2003 to evaluate national health promotion programs for risk of non-communicable diseases and was renamed the National Health and Nutrition Survey (NHNS). The NHNS is a cross-sectional survey conducted every November. The survey uses a stratified two-stage cluster sample design, and the sampling frame is the list of all residential census enumeration areas stratified into 47 prefectures. For the first sampling stage, a simple random sample of census enumeration areas is drawn from each prefecture. In the second sampling stage, selected census enumeration areas are divided into unit blocks so that each block consists of 20 to 30 households, and unit blocks are sampled using a simple random sampling from each prefecture. All individuals aged 1 year and older living in private households in 300 sampled unit blocks are eligible to participate in the survey.

In 1995, the survey started using single-day, semi-weighed household dietary records to assess dietary intake of individuals aged 1 year or older. Food intake is recorded on a single day in November, excluding Sundays and public holidays. Dietary records are weighed by taking inventory of all food and beverages consumed by a household and assigning approximate proportions of each item to individual household members. Prior to the survey, trained interviewers visit each household to distribute a self-administered questionnaire on food intake and instruct household representatives, who are typically responsible for food preparation, to measure quantities of food and beverages and complete the questionnaire. Household representatives are instructed to use a scale to weigh each food item and beverage consumed in the household and record this information, in addition to the allocation of shared dishes across individual household members on the questionnaire. In addition, food waste, leftovers, and food eaten away from home are also recorded. When survey interviewers visit households to collect the questionnaires, they review the questionnaires with the household representatives to clarify entries, fill in any missing information, and correct any errors. The surveys used the standard tables of food composition in Japan to calculate nutrient intakes from dietary records, changing editions from the fourth^16^ to the fifth in 2001,^17^ the fifth revised and enlarged in 2005,^18^ and the 2010 edition in 2011.^19^ A previous study estimated that the change in mean energy intake through revisions from the fourth to the fifth editions was less than 2%.^20^ We therefore considered that the continuity of estimated mean energy intake was retained across the revisions.

The survey also asks that all participants aged 1 year and older undergo a physical examination at a designated facility within walking distance of their residence during the survey period. In the physical examination, trained interviewers take anthropometric measurements and ask interview respondents about their concurrent medications and physical activity. Adult participants are further requested to have a venous blood sample taken. The survey introduced a blood test as part of the physical examination for participants aged 30 years in 1989, and the minimum age of eligibility was lowered to 20 years in 1997. For members of households not handing in the food intake questionnaire to survey staff at the time of collection, the physical examination is the only in-person opportunity to review and correct the dietary records.

### Statistical analysis

We applied a linear mixed-effects model separately by age groups (20–64 and 65–89 years) and sex to examine whether total energy intake was associated with the lack of an in-person review of dietary records. The random effects entered into the model at the levels of survey years, prefectures, unit blocks, and households to account for the analytical design of pooling data across survey years and the complex survey sampling design, including stratification and clustering in each survey year. In the model, the dependent variable was total energy intake of individual respondents (in kilocalories), and independent variables were 5-year age groups, body mass index (BMI; missing, <18.5, 18.5–24.9, 25.0–29.9, and ≥30.0 kg/m^2^), indicator variables on living in a single-person household and buying lunch, and a proxy variable for participation in a physical examination. We assumed that respondents participated in a physical examination and reviewed dietary records with a survey interviewer if they had a blood test recorded, because blood samples were drawn only at the physical examination site. We considered that respondents were tested if at least one value of hemoglobin, serum total cholesterol, glucose, and hemoglobin A_1c_ was valid, because these four items covered three blood collection tubes used in the survey. We subsequently classified respondents into three groups: A, respondents having at least one valid item from their blood test (reference group); B, respondents not having any valid item from their blood test but having one from other household members; and C, respondents having no valid items from their blood test or those of household members. We regarded group A as participants in the physical examination, group B as nonparticipants having a participant in the physical examination from their household, and group C as nonparticipants who had no household participants in the physical examination.

We assumed regression coefficients on nonparticipation in a physical examination adjusted for confounding with covariates to be negative and represent average underestimation of total energy intake due to the lack of an in-person review of dietary records. We added absolute values of these coefficients to the total energy intake of respondents in groups B and C, in order to correct their total energy intake. We then estimated mean energy intake from the corrected total energy intake under a counterfactual scenario (ie, all respondents participated in a physical examination and completed an in-person review of dietary records). We compared the corrected estimates of mean energy intake with crude means estimated from observed data by age group, sex, and three-year period. We used Wald tests adjusted for complex survey designs to test the equality of crude and corrected means of energy intake.

We applied a two-sample test of proportions and a two-sample *t* test to assess the equality of proportions and means, respectively, between 1997–1999 and 2009–2011. We used a chi-square test and one-way analysis of variance to test the equality of proportions and means, respectively, across groups defined by the status of participation in a physical examination in 1997–2011. We performed all analyses with STATA/MP 13.1 (StataCorp LP, College Station, TX, USA).

## RESULTS

In the pooled sample of the surveys from 1997 to 2011, participants who underwent a physical examination (group A) accounted for more than 60% of men and women aged 65–89 years, while these respondents accounted for 43% of men aged 20–64 years (Table 1). Nonparticipants with participants in their household (group B) accounted for 31% of men aged 20–64 years, while the proportions ranged from 13% to 17% in other sex and age groups. The proportion of respondents in group A significantly decreased by 3 to 9 percentage points in all sex and age groups between the 1997–1999 and 2009–2011 periods, while that of respondents with no participating household members (group C) significantly increased by 5 to 6 percentage points during the same span. The proportion of respondents in group B remained stable among men, while in women, it significantly increased for ages 20–64 years and decreased for ages 65–89 years.

**Table 1.** Distribution of study subjects by the status of participation in a physical examination, sex, age group, and 3-year survey period

Sex, age, participation in a physical examination	1997–2011		1997–1999		2000–2002		2003–2005		2006–2008		2009–2011		Difference between 1997–1999 and 2009–2011	
						
	*n*	(%)	*n*	(%)	*n*	(%)	*n*	(%)	*n*	(%)	*n*	(%)	Percentage points^a^	*P*-value
Men														
20–64 years														
A	18 361	(42.9)	5128	(45.4)	4392	(43.6)	3162	(40.9)	2988	(42.1)	2691	(40.6)	−4.8	<0.001
B	13 339	(31.1)	3419	(30.3)	3142	(31.2)	2521	(32.6)	2271	(32.0)	1986	(30.0)	−0.3	0.681
C	11 133	(26.0)	2755	(24.4)	2540	(25.2)	2045	(26.5)	1841	(25.9)	1952	(29.4)	5.1	<0.001
65–89 years														
A	10 232	(66.2)	2079	(67.8)	2140	(67.5)	1949	(66.2)	2105	(65.9)	1959	(63.6)	−4.2	0.001
B	2673	(17.3)	553	(18.0)	550	(17.4)	495	(16.8)	560	(17.5)	515	(16.7)	−1.3	0.172
C	2552	(16.5)	435	(14.2)	479	(15.1)	501	(17.0)	529	(16.6)	608	(19.7)	5.5	<0.001
Women														
20–64 years														
A	29 637	(61.4)	8199	(64.6)	7116	(63.0)	5249	(60.6)	4874	(60.0)	4199	(55.8)	−8.8	<0.001
B	6505	(13.5)	1549	(12.2)	1406	(12.4)	1202	(13.9)	1198	(14.7)	1150	(15.3)	3.1	<0.001
C	12 163	(25.2)	2944	(23.2)	2781	(24.6)	2210	(25.5)	2058	(25.3)	2170	(28.9)	5.7	<0.001
65–89 years														
A	13 202	(67.3)	2827	(69.3)	2724	(67.3)	2506	(67.1)	2563	(67.0)	2582	(65.9)	−3.4	0.001
B	2674	(13.6)	560	(13.7)	586	(14.5)	524	(14.0)	533	(13.9)	471	(12.0)	−1.7	0.022
C	3730	(19.0)	691	(16.9)	736	(18.2)	707	(18.9)	730	(19.1)	866	(22.1)	5.2	<0.001

A: Participants; B: Nonparticipants with a participant living in their household; C: Nonparticipants with no participants in their household.

^a^Equivalent to % in 2009–2011 minus % in 1997–1999.

Mean energy intake in 1997–2011 was significantly different across groups A, B, and C for all sex and age groups (Table 2). It was highest for group A in all sex and age groups, while it was lowest for group C in men and for group B in women. Mean energy intake significantly decreased in all three groups by approximately 140 to 210 kcal for both sexes aged 20–64 years from the 1997–1999 to the 2009–2011 survey periods. In the 65- to 89-years age group, the decrease was significant only among respondents in group C in men and group A in women.

**Table 2.** Mean energy intake of study subjects by status of participation in a physical examination, sex, age group, and 3-year survey period

Sex, age, participation in a physical examination	Mean energy intake, kcal (standard deviation)						Difference between 1997–1999 and 2009–2011	
	
	1997–2011	1997–1999	2000–2002	2003–2005	2006–2008	2009–2011	kcal^b^	*P*-value
Men								
20–64 years								
A	2304 (604)^a^	2373 (602)	2318 (603)	2281 (612)	2253 (598)	2230 (591)	−144	<0.001
B	2189 (620)	2258 (635)	2200 (607)	2183 (639)	2146 (602)	2110 (599)	−149	<0.001
C	2135 (629)	2262 (651)	2131 (620)	2093 (596)	2082 (633)	2054 (611)	−207	<0.001
65–89 years								
A	2078 (548)^a^	2073 (555)	2088 (554)	2090 (559)	2092 (548)	2045 (519)	−27	0.106
B	1924 (581)	1926 (602)	1911 (585)	1966 (602)	1899 (562)	1922 (553)	−4	0.920
C	1898 (556)	1930 (595)	1894 (555)	1907 (560)	1913 (557)	1857 (523)	−73	0.035
Women								
20–64 years								
A	1814 (471)^a^	1875 (487)	1831 (467)	1797 (474)	1769 (461)	1738 (440)	−136	<0.001
B	1699 (492)	1796 (523)	1702 (499)	1660 (495)	1676 (469)	1628 (440)	−168	<0.001
C	1733 (496)	1847 (529)	1753 (486)	1689 (478)	1689 (478)	1637 (465)	−210	<0.001
65–89 years								
A	1710 (454)^a^	1710 (466)	1731 (476)	1717 (463)	1708 (441)	1681 (417)	−29	0.018
B	1537 (449)	1526 (486)	1554 (468)	1550 (456)	1537 (422)	1516 (397)	−9	0.736
C	1584 (462)	1591 (449)	1602 (488)	1598 (488)	1577 (436)	1556 (449)	−34	0.133

A: Participants; B: Nonparticipants with a participant living in their household; C: Nonparticipants with no participants in their household.

^a^*P* < 0.001 for the test of the equality of means across groups in 1997–2011.

^b^Equivalent to the mean in 2009–2011 minus the mean in 1997–1999.

As for covariates, the proportion of respondents living in a single-person household was 0% for the group B respondents and was significantly higher for the group C respondents compared with group A respondents in all sex and age segments (Table 3). The proportions of respondents buying lunch were significantly different among groups A, B, and C in all sex and age segments. The proportion of respondents buying lunch was higher for respondents in groups B and C compared with those in group A and was higher for men aged 20–64 years compared with any other sex or age segment. BMI was missing for 33% to 46% of respondents in groups B and C; a substantial portion of nonparticipants in a physical examination who had valid BMI were assumed to have self-reported height and weight at home. The distribution of valid BMI differed significantly among groups A, B, and C for all sex and age segments.

**Table 3.** Characteristics of study subjects by status of participation in a physical examination, sex, and age group, from pooled data of surveys in 1997–2011

Sex, age, participation in a physical examination	Living in a single-person household	Buying lunch	Body mass index (kg/m^2^)						
		
	*n* (%)	*n* (%)	Missing	Valid	<18.5	18.5–24.9	25.0–29.9	≥30.0

			*n*	*n*	*n* (%)^c^	*n* (%)^c^	*n* (%)^c^	*n* (%)^c^
Men								
20–64 years								
A	1571 (8.6)^a^	6255 (34.1)^b^	11	18 350	623 (3.4)	11 861 (64.6)	5140 (28.0)	726 (4.0)^b^
B	0 (0.0)	5781 (43.3)	5344	7995	422 (5.3)	5543 (69.3)	1785 (22.3)	245 (3.1)
C	1217 (10.9)	5408 (48.6)	3853	7280	314 (4.3)	5177 (71.1)	1553 (21.3)	236 (3.2)
65–89 years								
A	901 (8.8)^a^	1709 (16.7)^b^	17	10 215	556 (5.4)	6798 (66.5)	2688 (26.3)	173 (1.7)^b^
B	0 (0.0)	482 (18.0)	1200	1473	158 (10.7)	1038 (70.5)	254 (17.2)	23 (1.6)
C	323 (12.7)	569 (22.3)	906	1646	150 (9.1)	1175 (71.4)	291 (17.7)	30 (1.8)
Women								
20–64 years								
A	1552 (5.2)^a^	6817 (23.0)^b^	37	29 600	2681 (9.1)	21 008 (71.0)	4875 (16.5)	1036 (3.5)^b^
B	0 (0.0)	2142 (32.9)	2575	3930	709 (18.0)	2705 (68.8)	400 (10.2)	116 (3.0)
C	909 (7.5)	3832 (31.5)	4067	8096	1052 (13.0)	5832 (72.0)	1023 (12.6)	189 (2.3)
65–89 years								
A	2737 (20.7)^a^	1787 (13.5)^b^	28	13 174	903 (6.9)	8326 (63.2)	3384 (25.7)	561 (4.3)^b^
B	0 (0.0)	415 (15.5)	1232	1442	195 (13.5)	902 (62.6)	283 (19.6)	62 (4.3)
C	963 (25.8)	658 (17.6)	1221	2509	281 (11.2)	1620 (64.6)	528 (21.0)	80 (3.2)

A: Participants; B: Nonparticipants with a participant living in their household; C: Nonparticipants with no participants in their household.

^a^*P* < 0.001 for the test of the equality of proportions between the groups A and C.

^b^*P* < 0.001 for the test of the equality of proportions across groups A, B, and C.

^c^Percentage out of valid cases.

Adjusted for confounding with these covariates, total energy intake was significantly inversely associated with non-participation of respondents in a physical examination across all sex and age groups, irrespective of the participation of other household members (Table 4). In men, the absolute value of the association was significantly smaller for respondents in group B compared with those in group C, by 45 kcal for those aged 20–64 years (*P* < 0.001) and 39 kcal for those aged 65–89 years (*P* = 0.010). However, this difference was not significant for women (*P* = 0.351 for those aged 20–64 years and *P* = 0.898 for those aged 65–89 years).

**Table 4.** Regression coefficients of total daily energy intake on the status of participation in a physical examination, by age group and sex, using the pooled data of National Nutrition Surveys 1997–2002 and National Health and Nutrition Surveys 2003–2011

Sex, age	*n*	Status of participation in a physical examination				

		A	B		C	
	
			β coefficient	(SE)	β coefficient	(SE)
Men						
20–64 years	42 833	Reference	−82.2	(8.0)*	−127.2	(8.4)*
65–89 years	15 457	Reference	−98.9	(13.7)*	−138.1	(13.3)*
Women						
20–64 years	48 305	Reference	−67.6	(7.2)*	−60.6	(6.0)*
65–89 years	19 606	Reference	−98.7	(11.1)*	−97.2	(9.3)*

SE, standard error.

A: Participants; B: Nonparticipants with a participant living in their household; C: Nonparticipants with no participants in their household.

Estimates are adjusted for 5-year age groups, living in a single-person household, buying lunch, and body mass index.

*P < 0.001.

We estimated corrected mean total energy intake under the counterfactual scenario that all respondents participated in a physical examination. After correction, mean total energy intake significantly increased by 1% to 3% across the study period compared to means estimated from observed data in all sex and age groups (Table 5). The decreasing secular trend in mean energy intake from 1997–1999 to 2009–2011 was sustained after correction, at approximately 160 to 170 kcal in both sexes aged 20–64 years.

**Table 5.** Observed and corrected means of total daily energy intake in the Japanese population, by age group, sex, and 3-year survey period

Sex, age, survey year	Mean energy intake, kcal (standard error)		

	Observed	Corrected	Corrected-Observed
Men			
20–89 years			
1997–1999	2222 (5)	2273 (5)	51 (1.1)*
2000–2002	2167 (6)	2220 (6)	53 (0.9)*
2003–2005	2145 (7)	2200 (7)	56 (0.9)*
2006–2008	2127 (7)	2182 (7)	55 (0.9)*
2009–2011	2096 (7)	2154 (7)	58 (0.9)*
20–64 years			
1997–1999	2312 (6)	2367 (6)	55 (1.2)*
2000–2002	2232 (7)	2290 (7)	58 (1.0)*
2003–2005	2195 (8)	2257 (8)	61 (1.1)*
2006–2008	2171 (9)	2231 (9)	60 (0.9)*
2009–2011	2141 (8)	2204 (8)	63 (0.9)*
65–89 years			
1997–1999	1981 (10)	2021 (10)	39 (1.4)*
2000–2002	1991 (10)	2031 (9)	40 (1.3)*
2003–2005	2008 (11)	2049 (11)	41 (1.2)*
2006–2008	2008 (11)	2049 (11)	41 (1.3)*
2009–2011	1974 (9)	2018 (9)	44 (1.3)*
Women			
20–89 years			
1997–1999	1800 (5)	1825 (5)	25 (0.5)*
2000–2002	1759 (5)	1785 (4)	26 (0.6)*
2003–2005	1722 (5)	1750 (5)	27 (0.5)*
2006–2008	1710 (5)	1738 (5)	28 (0.5)*
2009–2011	1672 (5)	1702 (5)	30 (0.5)*
20–64 years			
1997–1999	1855 (5)	1877 (5)	22 (0.5)*
2000–2002	1791 (5)	1814 (5)	24 (0.5)*
2003–2005	1745 (5)	1770 (5)	25 (0.5)*
2006–2008	1730 (6)	1757 (6)	26 (0.5)*
2009–2011	1686 (6)	1715 (6)	29 (0.5)*
65–89 years			
1997–1999	1651 (8)	1683 (8)	32 (0.9)*
2000–2002	1675 (8)	1708 (8)	33 (1.0)*
2003–2005	1662 (8)	1695 (8)	33 (0.9)*
2006–2008	1655 (7)	1687 (7)	33 (0.9)*
2009–2011	1634 (7)	1667 (7)	33 (1.0)*

Estimates are adjusted for age using the population of Japan in 2010.^25^

*P < 0.001.

## DISCUSSION

To our knowledge, this is the first study to investigate the association of total energy intake with the lack of an in-person review of semi-weighed household dietary records in the NHNS. Our results demonstrated that total energy intake of respondents who did not participate in a physical examination was significantly lower compared with respondents who did. This finding suggests that total energy intake may be underestimated without an in-person review on site and that further efforts to facilitate participation of respondents in a physical examination may help improve accuracy of these data.

Our results also indicated the possibility that, particularly in men, a review of dietary records by other household respondents on behalf of respondents who are absent from a physical examination might increase the accuracy of total energy intake. However, the reviews of dietary information by surrogate respondents may have only a limited impact, because other household respondents may be unable to adequately probe for portion size of food bought for lunch outside of the home on weekdays, such as the amount of boiled rice. In Japan, approximately 40% of working-age men eat lunch outside of their homes, and rice accounts for approximately 30% of total caloric intake per capita.^3^ In the NHNS, household representatives record only the name of dishes and the number of servings for meals bought outside of the home and they do not have to provide details about the portion size of individual food and drink items. For female respondents who did not have a physical examination, a proxy review had little additional effect on correcting total energy intake, indicating that other household members may not be aware of their diet. Therefore, to improve the accuracy of data on total energy intake, we recommend that respondents participate in a physical examination and review dietary records on their own rather than relying on the reports of other household members.

In the NHNS, the proportion of respondents receiving a physical examination has been gradually decreasing. This trend is largely attributable to the decrease in participation of respondents from households with two or more persons, a group that accounts for approximately 90% of the sample used in our analysis. However, our additional analysis showed that the proportion of households with two or more persons and with no household member participating in a physical examination has increased steadily from 19% in 1997 to 32% in 2011, while the rate of single-person households not participating in a physical examination has fluctuated between 30% and 40% (data not shown). Therefore, encouraging participation of households with two or more persons in a physical examination would be an important strategy for decreasing underreporting of total energy intake in the NHNS.

At the population level, if the proportion of respondents having a review of dietary intake records at a physical examination increased in the NHNS, mean energy intake would be expected to increase based on values from our crude data. However, these changes would account for less than 5% of mean energy intake before correction, even if all respondents received a physical examination. This may appear to be a rather small proportion compared with past results suggesting differences of 10% to 20% between estimated total energy expenditure and reported energy intake in weighed dietary records.^21^ A study of National Health and Nutrition Examination Surveys in the United States demonstrated that the mean difference between total energy intake reported during a 24-hour dietary recall interview and estimated total energy expenditure was, at most, 10% in men and 18% in women.^10^ Another study that analyzed 7-day weighed dietary records from the 2000 National Diet and Nutrition Survey in the United Kingdom found that the median difference between reported energy intake and estimated energy requirements was nearly 30%.^22^ The present study may not be directly comparable to these past studies based on the principle of energy balance, but the results suggest that the contribution of the lack of in-person reviews at a physical examination site to underestimation of mean energy intake may be limited in the NHNS. In addition, the increasing proportion of respondents not participating in a physical examination was not sufficient to offset the decreasing trend in mean energy intake. Other potential factors for decreased caloric intake to be explored in future studies might include an increase in the proportion of individuals skipping breakfast.^3^

Our analysis has some limitations. First, we did not examine the effects on total energy intake of a review of dietary records at home, as it was not possible to identify which respondents had undergone a review when survey interviewers visited their homes. This might be particularly concerning for respondents who had difficulty leaving home without assistance, who account for approximately 8% of the older population.^23^ The absolute magnitude of the association between total energy intake and the lack of in-person reviews might be expected to increase if a review in a survey respondent’s home was considered. However, we believe that participation in a physical examination likely covered the majority of physically mobile respondents who underwent a home review, as those staying at home to meet interviewers might be motivated to undergo a physical examination as well. Second, no direct measure was available to assess an in-person review, because the survey did not record whether individual respondents underwent a review of dietary records. However, we believed that use of a proxy variable was the best possible solution given the constraint of data availability. Third, we did not have a biomarker to directly assess underestimation of total energy intake for individual respondents but only obtained the average population estimates by age group and sex. Previous studies assessed misreporting of total energy intake for individual respondents in national surveys using biomarkers for validation, such as basal or resting metabolic rate^10^^,^^11^^,^^13^^,^^14^ and total energy requirements,^10^^,^^12^^,^^22^ which require height and body weight for estimation. We did not examine these biomarkers because anthropometric data were missing for respondents who missed a physical examination. Fourth, the present study did not consider reporting bias in energy intake associated with reactivity, such as social desirability and changes in eating behavior. Previous studies have revealed that these effects are important for underreporting, particularly among women and individuals with high body weight.^24^ However, we believe that a single day of recording was likely short enough to prevent reactivity.

In conclusion, an in-person review of semi-weighed household dietary records at a physical examination site may decrease underestimation of total energy intake in the NHNS. Further strategies to promote participation of respondents in a physical examination, particularly from households with two or more persons, may be needed to ensure completion of a review of dietary records and improve the accuracy of data on total energy intake. However, the gradual increase in the proportion of respondents missing an in-person review contributed little to the decrease in mean total energy intake at the population level. Further research is needed to explore factors that have contributed to the downward trend of caloric intake in Japan.

## ONLINE ONLY MATERIAL

Abstract in Japanese.
