# Supplementary material for: Association of Energy Intake With the Lack of in-Person Review of Household Dietary Records: Analysis of Japan National Health and Nutrition Surveys From 1997 to 2011
Source: J Epidemiol. 2016 Feb 5;26(2):84–91. doi: 10.2188/jea.JE20150048 (PMC4728119; doi:10.2188/jea.JE20150048)
Supplement: Abstract in Japanese. [file je-26-084-s001.pdf]

平成9年～平成23年国民健康・栄養調査における世帯単位の食事記録に関する対面確認の不足とエネルギー摂取量との関連

見出し表題: 総エネルギー摂取量と世帯単位の食事記録における対面確認の不足

池田奈由[1]、 奥田奈賀子[2]、坪田(宇津木)恵[1]、西　信雄[1]

（１）国立健康・栄養研究所　国際産学連携センター
（２）人間総合科学大学　健康栄養学科

要約
背景 : 国民健康・栄養調査の結果によると、平均エネルギー摂取量は長期に渡り低下傾向にあるが、過体重と肥満の有病率の減少傾向は示されていない。本研究は、国民健康・栄養調査において調査員と被調査者が対面で行う食事記録の確認の有無が、総エネルギー摂取量と関連するか、また平均エネルギー摂取量の減少傾向に影響を与えているかについて検討した。

方法 : 国民栄養調査（平成9年～平成14年）および国民健康・栄養調査（平成15年～平成23年）の20歳から89歳の個人のデータを統合した。総エネルギー摂取量と、半定量法による世帯単位の食事記録に関する被調査者との対面確認が欠落していることとの関連について、線形混合効果モデルによる推定を行った。食事記録が確認されていない被調査者については、総エネルギー摂取量を回帰係数で調整した。

結果 : 対面確認を完了した被調査者と比較して、確認を完了していない被調査者の総エネルギー摂取量は、すべての性・年齢階級において有意な負の相関を示した（P値<0.001）。対面確認を完了していない被調査者の総エネルギー摂取量の補正により、各調査年における平均エネルギー摂取量は男性では2.1%から3.9%、女性では1.3%から2.6%、有意に上昇したが（P値<0.001）、平均エネルギー摂取量の減少傾向には変化がなかった。

結論: 食事記録の対面確認を完了していないことにより、総エネルギー摂取量は過少に推定される可能性がある。今後、対面確認の完了を促進する取り組みによって、これらデータの精度の向上が見込まれる。一方で、対面確認を完了していない被調査者の割合の増加は、平均エネルギー摂取量の減少にほとんど影響を与えていない可能性が示唆された。

Key words: 総エネルギー摂取量、国民健康・栄養調査、半定量法による食事記録、対面確認
